# Systematic Analysis of Transcriptomic Profile of Chondrocytes in Osteoarthritic Knee Using Next-Generation Sequencing and Bioinformatics

**DOI:** 10.3390/jcm7120535

**Published:** 2018-12-10

**Authors:** Yi-Jen Chen, Wei-An Chang, Ling-Yu Wu, Ya-Ling Hsu, Chia-Hsin Chen, Po-Lin Kuo

**Affiliations:** 1 Graduate Institute of Clinical Medicine, College of Medicine, Kaohsiung Medical University, Kaohsiung 807, Taiwan; chernkmu@gmail.com (Y.-J.C.); 960215kmuh@gmail.com (W.-A.C.); esther906@gmail.com (L.-Y.W.); 2 Department of Physical Medicine and Rehabilitation, Kaohsiung Medical University Hospital, Kaohsiung 807, Taiwan; 3 Division of Pulmonary and Critical Care Medicine, Kaohsiung Medical University Hospital, Kaohsiung 807, Taiwan; 4 Graduate Institute of Medicine, College of Medicine, Kaohsiung Medical University, Kaohsiung 807, Taiwan; hsuyl326@gmail.com; 5 Department of Physical Medicine and Rehabilitation, School of Medicine, College of Medicine, Kaohsiung Medical University, Kaohsiung 807, Taiwan; 6 Orthopaedic Research Center, Kaohsiung Medical University, Kaohsiung 807, Taiwan; 7 Institute of Medical Science and Technology, National Sun Yat-Sen University, Kaohsiung 804, Taiwan

**Keywords:** knee osteoarthritis, cartilage damage, chondrocytes, joint pain, next-generation sequencing, bioinformatics, microRNA, messenger RNA

## Abstract

The phenotypic change of chondrocytes and the interplay between cartilage and subchondral bone in osteoarthritis (OA) has received much attention. Structural changes with nerve ingrowth and vascular penetration within OA cartilage may contribute to arthritic joint pain. The aim of this study was to identify differentially expressed genes and potential miRNA regulations in OA knee chondrocytes through next-generation sequencing and bioinformatics analysis. Results suggested the involvement of SMAD family member 3 (*SMAD3*) and Wnt family member 5A (*WNT5A*) in the growth of blood vessels and cell aggregation, representing features of cartilage damage in OA. Additionally, 26 dysregulated genes with potential miRNA–mRNA interactions were identified in OA knee chondrocytes. Myristoylated alanine rich protein kinase C substrate (*MARCKS*), epiregulin (*EREG*), leucine rich repeat containing 15 (*LRRC15*), and phosphodiesterase 3A (*PDE3A*) expression patterns were similar among related OA cartilage, subchondral bone and synovial tissue arrays in Gene Expression Omnibus database. The Ingenuity Pathway Analysis identified *MARCKS* to be associated with the outgrowth of neurite, and novel miRNA regulations were proposed to play critical roles in the pathogenesis of the altered OA knee joint microenvironment. The current findings suggest new perspectives in studying novel genes potentially contributing to arthritic joint pain in knee OA, which may assist in finding new targets for OA treatment.

## 1. Introduction

Osteoarthritis (OA) is one of the common musculoskeletal disorders with increased incidence in the elderly [1]. The diagnosis of OA is based on clinical symptoms and radiographic findings, such as narrowed joint space and irregular cortical surface with the presence of osteophytes; the clinical symptoms of OA include pain, swelling and joint deformity that impede normal walking [2,3]. The cardinal pathological change in the OA joint is the destruction of articular cartilage. During degeneration and OA changes, chondrocytes shift towards a hypertrophic phenotype, and proliferation and hypertrophic differentiation of chondrocytes occur, with the characteristics of decreased synthesis of the extracellular matrix (ECM) and increased production of proteolytic enzymes such as metalloproteinases (MMPs), resulting in remodeling and mineralization of ECM [4,5]. Hypertrophic chondrocytes also promote angiogenesis in OA [6]. 

The biological functions of chondrocytes and histopathological changes of articular cartilage in OA have been extensively studied [5,7]. While cartilage breakdown is the major consequence of OA, there is a growing consensus that OA is considered a disease of the entire joint, affecting all tissues in the joint, including cartilage, subchondral bone, meniscus, ligament, and synovium, and is not merely a process of wear and tear [8]. Of note, the interplay between cartilage and subchondral bone, mainly mediated by chondrocytes and osteoblasts within the two compartments, has received much attention and potentially contributes to the pathogenesis of OA [4]. The abnormal remodeling and increased vascularization observed in the OA joint facilitate molecular transport and cellular interaction between cartilage and bone [9].

MicroRNAs (miRNAs) are small non-coding single-stranded RNAs that regulate gene expression through binding to the 3’ untranslated region (UTR) of target mRNAs [10]. The functions of miRNAs have been shown to play critical roles in the pathogenesis of OA, regulating the gene expressions of several proteolytic enzymes and influencing various growth factor signaling pathways [10,11,12]. The advanced technology of high-throughput next-generation sequencing (NGS) has made available a rapid gain of a large volume of genomic data [13], and the use of bioinformatics approaches to analyze these large datasets gives better understanding and more rapid analysis of associations and biological functions of selected lists of genes and/or miRNAs [14,15,16]. In the current study, different bioinformatics databases and tools were used to recognize biological functions and potential miRNA–mRNA interactions involved in OA chondrocytes, including Ingenuity Pathway Analysis (IPA) [17], Database for Annotation, Visualization and Integrated Discovery (DAVID) [18], Gene Expression Omnibus (GEO) [19], and miRmap [20,21]. Using the NGS and extensive bioinformatics analysis, we aim to identify novel miRNAs and potential miRNA regulations of target genes differentially expressed in OA chondrocytes. We expect the findings will provide new perspectives in the pathogenesis and potential therapeutic targets for patients with OA.

## 2. Materials and Methods

### 2.1. Cell Culture

Chondrocytes of normal adult and osteoarthritic knee cartilage were purchased from Cell Applications, Inc. (San Diego, CA, USA), which were cryopreserved at the first passage. Chondrocytes were cultured in Chondrocyte Growth Medium (Cell Applications, Inc. San Diego, CA, USA) and placed in a 37 °C, 5% CO_2_ humidified incubator until confluence, at 7 days after seeding. The normal and OA knee chondrocytes were then harvested for total RNA extraction and further expression profiling of mRNA and small RNA.

### 2.2. RNA Sequencing

Total RNAs of normal and OA knee chondrocytes were extracted using Trizol Reagent (Invitrogen, Carlsbad, CA, USA) following the manufacturer’s instructions and the quality of extracted RNAs were analyzed for OD_260_ detection by ND-1000 spectrophotometer (Nanodrop Technology, Wilmington, DE, USA) before sequencing. The quality of extracted RNAs were validated by RNA integrity number (RIN) with Agilent Bioanalyzer (Agilent Technology, Santa Clara, CA, USA), with RIN of 9.8 for normal knee chondrocytes and 9.9 for OA knee chondrocytes. The sequencing analysis for RNA and small RNA were carried out by Welgene Biotechnology Company (Welgene, Taipei, Taiwan). For RNA sequencing, the library preparation and sequencing procedures were carried out following the manufacture’s protocol from Illumina. A read length of 75 nucleotides single-end sequencing on Solexa platform was performed for library construction, the sequence was determined using sequencing-by-synthesis technology, and raw sequences obtained from Illumina Pipeline software bcl2fastq v2.0 generated 30 million reads per sample. The raw sequences were filtered and trimmed for qualified reads, and TopHat/Cufflinks method was used for gene expression estimation. The expression level was normalized by calculating fragments per kilobase of transcript per million mapped reads (FPKM). The reference genome and gene annotations were obtained from Ensembl database. For small RNA sequencing, library construction was prepared using Illumina sample preparation kit, following the TruSeq Small RNA Sample Preparation Guide. The 3′ and 5′ adaptors were ligated to total RNA, reverse transcribed, and PCR amplified. The cDNA constructs were fractionated and purified for bands containing 18–40 nucleotide RNA fragments. The prepared libraries were sequenced with a read length of 75 nucleotides single-end and processed with Illumina instrument and software. The raw sequences were filtered and trimmed for qualified reads, analyzed in miRDeep2 to clip 3’ adaptor sequence, and aligned to the human genome from University of California, Santa Cruz (UCSC). All reads were normalized by Reads Per Million (RPM) [22]. The miRDeep2 was used to estimate expression levels of known and novel miRNAs. The sequencing quality for both RNA and small RNA was determined by FastQC, a quality control tool for high-throughput sequence data, reporting per-base sequence quality and per-sequence quality scores. The criteria of differentially expressed mRNAs and miRNAs were set at fold change >2.0, FPKM >0.3 for mRNA and RPM >10 for miRNA. The threshold of FPKM >0.3 for mRNA was selected, since lower confidence in measured expression levels below 0.3 was reported [23]. The threshold of RPM >10 for miRNA was selected to identify functional miRNAs as previously reported [24]. 

### 2.3. Ingenuity Pathway Analysis (IPA)

The Ingenuity Pathway Analysis (IPA) software (Ingenuity systems, Redwood City, CA, USA) provides bioinformatics analysis of gene arrays; RNA and small RNA sequencing, proteomics and other biological experiments; categorizes molecules into different biological networks; and identifies related canonical pathways, upstream regulators, diseases and functions involved in a subset of molecules. The IPA core analysis provides predicted canonical pathways based on the literature and *de novo* network discovery based on expression changes of uploaded experimental results [17]. Additionally, construction of causal networks based on gene interactions curated from the literature is also available, which provides mechanistic hypotheses based on expression changes of the uploaded dataset. The statistical significance of the constructed regulatory networks was determined by Fisher’s exact test *p*-value (enrichment score) that measured the overlap of observed and predicted gene sets, and Z score that measured the match of observed and predicted regulatory patterns with directional effect [25]. In the current study, differentially expressed genes with fold changes in expression between normal and OA knee chondrocytes were uploaded into IPA for core analysis to obtain pathway and network results. For “create expression analysis” setting, we took into consideration all direct and indirect relationships identified in all data sources with experimentally observed or moderate to highly predicted confidence, without specifying tissue types or species.

### 2.4. Database for Annotation, Visualization and Integrated Discovery (DAVID) Bioinformatics Resources

The DAVID bioinformatics resources is an open-source bioinformatics database that provides an integrated data-mining environment and categorizes a list of genes into different biological functions using different tools such as functional annotation and gene functional classification. Users are able to have an overall concept of the biological themes that are enriched in the list of genes [18]. Differentially expressed genes of interest were input into DAVID database for functional annotation analysis, and the Expression Analysis Systematic Explorer (EASE) score was set at a default cutoff value of 0.1, representing the modified Fisher’s exact *p*-value in the DAVID database.

### 2.5. Gene Expression Omnibus (GEO) Database

The GEO database provides high-throughput genomic data sets, and researchers can have access to the analysis of genes of interest and their expression values through web-based tools such as GEO2R, and perform further between-group comparisons [19]. The relevant datasets related to joint tissues of OA patients (GSE114007 for cartilages, GSE51588 for subchondral bones, GSE55235 and GSE55457 for synovial tissues) were used in this study to examine whether the expression patterns of genes of interest were in line with that observed in our expression profile of OA knee chondrocytes. Detailed information of the selected datasets was provided in .

### 2.6. miRmap Database

MiRmap is an open-source software library developed by Vejnar et al. that is available online. The library provides miRNA target prediction by ranking the repression strength of potential targets. The repression strength is predicted by a comprehensive approach, combining thermodynamic, evolutionary, probabilistic and sequence-based features, and is given a miRmap score for each potential target. The higher score indicates higher repression strength. In our current study, the 46 differentially expressed miRNAs were sequentially inputted into the library to obtain potential target genes. Those potential targets with miRmap scores higher than 99.0 were selected for further analysis [20,21].

### 2.7. Statistical Analysis

To determine differentially expressed mRNAs between normal and OA knee chondrocytes, statistical analysis was performed using Cuffdiff (Cufflinks version 2.2.1), with *p*-value calculation for non-grouped samples using blind mode, in which all samples were treated as replicates of a single global condition and used to blind one model for a statistic test [26]. Based on the selection of competitive null hypothesis and the use of over-representation analysis as the pathway analysis method for our pathway annotation analysis [16,27], we firstly selected from our high-throughput differential expression data those mRNAs with expression values of FPKM >0.3, a fold-change >2.0 and a *p*-value <0.05 between normal and OA knee chondrocytes. Further, multiple testing correction for *p*-value adjustments using Bonferroni adjustment method, Hochberg’s method and false discovery rate (FDR) controlling method [28,29] was performed using Statistical Analysis System (SAS) PROC MULTTEST (SAS version 9.4, SAS Institute, Cary, NC, USA) for the pre-selected differential expression gene list. An adjusted *p*-value of <0.05 indicated statistical significance. All pre-selected differential expression genes remained statistically significant after Hochberg’s method and FDR controlling method corrections. In addition, expression values of target genes in OA and non-OA patients identified from selected GEO arrays were analyzed for between-group differences, using IBM SPSS Statistics for Windows, version 19 (IBM Corp., Armonk, NY, USA). For the relatively small numbers of cases in each group, the non-parametric method with the Mann-Whitney *U* test was used for between-group comparison. A *p*-value < 0.05 was considered as a statistically significant difference.

## 3. Results

### 3.1. The Sequencing Quality and Mapped Reads for RNA and Small RNA Sequencing of Chondrocytes

The results of sequencing quality of RNA sequencing for normal and OA knee chondrocytes, using FastQC, are shown in Figure S1, reporting high quality scores in per-base sequence quality and per-sequence quality scores. Similarly, the quality scores of small RNA sequencing for normal and OA knee chondrocytes indicated good sequencing quality (Figure S2). The mapped reads for both RNA and small RNA sequencing are listed in Table S2. The raw sequencing data were uploaded to the GEO repository with the GEO accession number of GSE110606. The data will be held private before publication.

### 3.2. The Differentially Expressed Genes in Osteoarthritic Knee Chondrocytes were Associated with Osteoarthritis Pathway, Cell Adhesion and Extracellular Matrix Organization

**Figure 1:**
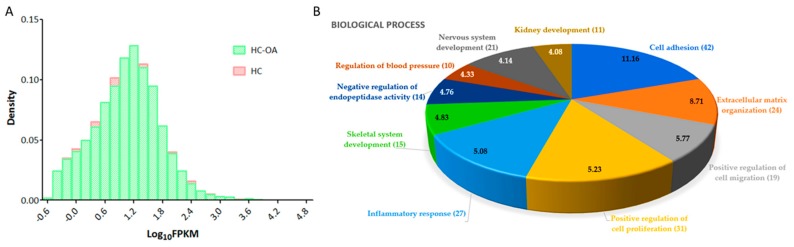
Differential expression analysis of genes in osteoarthritic (OA) knee chondrocytes. (**A**) For gene expressions with fragments per kilobase of transcript per million (FPKM) >0.3, the difference in FPKM performance between normal (HC) and OA (HC-OA) knee chondrocytes is shown in a density plot. The x-axis represents logarithm to the base 10 of FPKM, and the y-axis indicates read density. (**B**) The 495 differentially expressed protein-coding genes in OA knee chondrocytes were input into the DAVID database to determine related biological functions, and these dysregulated genes were related to cell adhesion (42 genes), extracellular matrix organization (24 genes), positive regulation of cell migration (19 genes), positive regulation of cell proliferation (31 genes), inflammatory response (27 genes), skeletal system development (15 genes), negative regulation of endopeptidase activity (14 genes), regulation of blood pressure (10 genes), nervous system development (21 genes), and kidney development (11 genes). The selected criteria for functional annotation analysis were Expression Analysis Systematic Explorer (EASE) score = 0.1 and fold enrichment >1.3. The −log (*p* value) of each biological term is indicated within the pie chart.

The expression values of normal and OA knee chondrocytes were obtained from the NGS results of RNA-sequencing and were further analyzed. Screening for gene expressions with FPKM >0.3, the difference in FPKM performance between normal and OA knee chondrocytes was displayed as a density plot, as indicated in Figure 1A. A total of 495 differentially expressed protein-coding genes with >2.0-fold-change and >0.3 FPKM were identified, where 263 genes were up-regulated and 232 genes were down-regulated in OA knee chondrocytes, compared to normal chondrocytes. The detailed information of the 495 differentially expressed genes is provided in Table S3. To determine the biological functions and pathways possibly involved in these differentially expressed genes in chondrocytes, we use the IPA software and the functional annotation tool in the DAVID database for further analysis. The results of IPA analysis identified the osteoarthritis pathway as the most highly enriched canonical pathway among these dysregulated genes, with 23 differentially expressed genes involved in this pathway (*p* = 4.72 × 10^−10^, Z score = 1.342), and tumor necrosis factor (*TNF*) as the most highly predicted upstream regulator (*p* = 2.20 × 10^−22^, Z score = −0.159). The top five canonical pathways and potential upstream regulators are listed in Table 1.

Through the functional annotation analysis in the DAVID database, the top 10 biological processes involved in these differentially expressed genes were identified to be related to cell adhesion (42 genes, *p* = 6.87 × 10^−12^, false discovery rate (FDR) = 1.22 × 10^−8^), extracellular matrix organization (24 genes, *p* = 1.94 × 10^−9^, FDR = 3.43 × 10^−6^), positive regulation of cell migration (19 genes, *p* = 1.71 × 10^−6^, FDR = 0.003), positive regulation of cell proliferation (31 genes, *p* = 5.95 × 10^−6^, FDR = 0.011), inflammatory response (27 genes, *p* = 8.25 × 10^−6^, FDR = 0.015), skeletal system development (15 genes, *p* = 1.47 × 10^−5^, FDR = 0.026), negative regulation of endopeptidase activity (14 genes, *p* = 1.73 × 10^−5^, FDR = 0.031), regulation of blood pressure (10 genes, *p* = 4.67 × 10^−5^, FDR = 0.083), nervous system development (21 genes, *p* =7.31 × 10^−5^, FDR = 0.130), and kidney development (11 genes, *p* = 8.24 × 10^−5^, FDR = 0.146), as shown in Figure 1B. The functional annotation clustering analysis of the 495 differentially expressed genes indicated the enrichment score of 2.47 for a group of genes related to biological functions of cartilage and bone. In addition, other significant biological functions (*p* < 0.05, fold enrichment >1.3) related to cellular, tissue and structural changes in OA joint, including regulation of cartilage development, chondrocyte differentiation and bone mineralization, endochondral ossification, and the corresponding differentially expressed genes are listed in Table 2. A total of nine up-regulated genes and six down-regulated genes in the OA knee chondrocytes were identified.

### 3.3. Identification of Dysregulated Genes Related to Joint Structural Damage in OA

**Figure 2:**
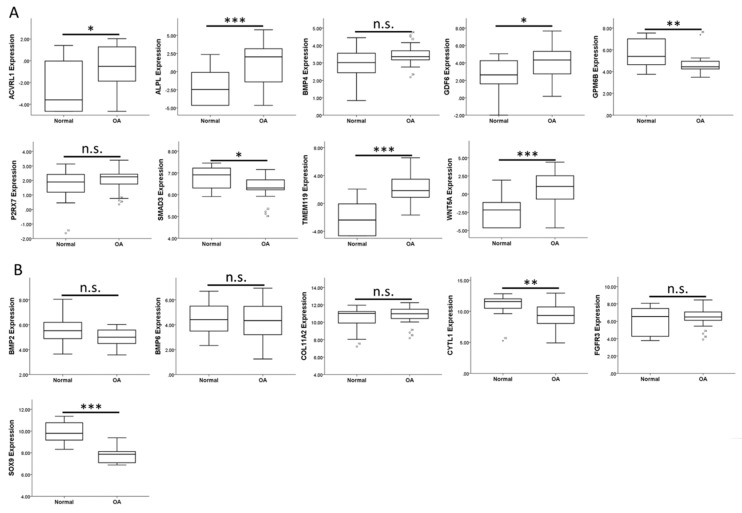
Expression patterns of genes related to the biological processes of OA in the GEO dataset of OA knee cartilages. The expression patterns of (**A**) 9 up-regulated genes and (**B**) 6 down-regulated genes related to biological processes of cellular, tissue and structural changes in OA were analyzed in a GEO dataset of normal and OA knee cartilages (GSE114007). *ACVRL1*, *ALPL*, *GDF6*, *TMEM119*, and *WNT5A* were significantly up-regulated, whereas *CYTL1* and *SOX9* were significantly down-regulated in OA knee cartilages. The expression patterns of *GPM6B* and *SMAD3* (down-regulated) in OA knee cartilage were different from that identified in our expression profile of OA knee chondrocytes (up-regulated). * indicated *p* < 0.05, ** indicated *p* < 0.01, *** indicated *p* < 0.001, and n.s. indicated no statistical significance between normal and OA groups.

**Figure 3:**
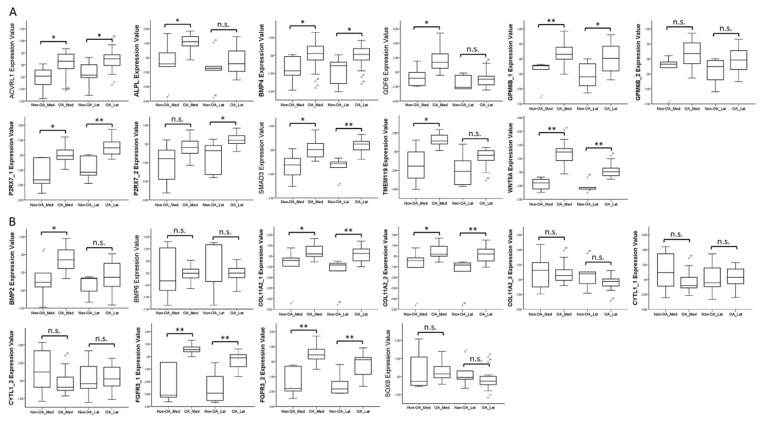
Expression patterns of genes related to biological processes of OA in GEO array of OA knee subchondral bone. The expression patterns of (**A**) nine up-regulated genes and (**B**) six down-regulated genes related to the biological processes of cellular, tissue and structural changes in OA were analyzed in a GEO array of normal and OA knee subchondral bone (GSE51588). *ACVRL1*, *BMP4*, *SMAD3*, and *WNT5A* were significantly up-regulated in both medial and lateral plateaus, whereas *ALPL*, *GDF6*, and *TMEM119* were significantly up-regulated only in the medial plateau of OA knees. The expression patterns of *BMP2*, *COL11A2*, and *FGFR3* (up-regulated) in subchondral bones of OA knees were different from that identified in our expression profile of OA knee chondrocytes (down-regulated). * indicated *p* < 0.05, ** indicated *p* < 0.01, and n.s. indicated no statistical significance between non-OA and OA groups. For those genes with more than one probe within the array, the expression patterns were considered consistent only if there were at least two probes with significant dysregulation in the same direction. (Probe ID reference: *ACVRL1*, A_24_P945113; *ALPL*, A_24_P353619; *BMP4*, A_23_P54144; *GDF6*, A_32_P140489; *GPM6B_1*, A_33_P3218649; *GPM6B_2*, A_23_P378416; *P2RX7_1*, A_24_P319113; *P2RX7_2*, A_33_P3263867; *SMAD3*, A_23_P48936; *TMEM119*, A_33_P3395605; *WNT5A*, A_33_P3341499; *BMP2*, A_33_P3237150; *BMP6*, A_23_P19624; *COL11A2_1*, A_33_P3216442; *COL11A2_2*, A_23_P42322; *COL11A2_3*, A_33_P3216448; *CYTL1_1*, A_33_P3252695; *CYTL1_2*, A_23_P10647; *FGFR3_1*, A_23_P500501; *FGFR3_2*, A_23_P212830; and *SOX9*, A_23_P26847).

To investigate the expression patterns of genes related to biological processes of cellular, tissue and structural changes in OA joint in clinical specimens, we searched in the GEO database for OA-related datasets. One RNA sequencing dataset consisting of 18 normal knee cartilages and 20 OA knee cartilages was found (GSE114007). One array comparing the expression profiles of subchondral bones at medial and lateral tibial plateaus of both normal and OA knee joints was also found (GSE51588). Considering the close contact of cartilage and subchondral bones and possible crosstalk between these joint tissues in the OA joint microenvironment, we simultaneously investigated the expression patterns of these genes in the subchondral bone dataset. The results showed that the expression patterns of *ACVRL1*, *ALPL*, *GDF6*, *TMEM119*, *WNT5A*, *CYTL1*, and *SOX9* in OA knee cartilages were consistent with our expression profile of OA chondrocytes (Figure 2A,B), while the expression patterns of *ACVRL1*, *ALPL*, *BMP4*, *GDF6*, *SMAD3*, *TMEM119*, and *WNT5A* in subchondral bones of OA knees were consistent with our data (Figure 3A,B).

### 3.4. SMAD3 and WNT5A were Involved in Growth of Blood Vessel and Cell Aggregation

**Figure 4:**
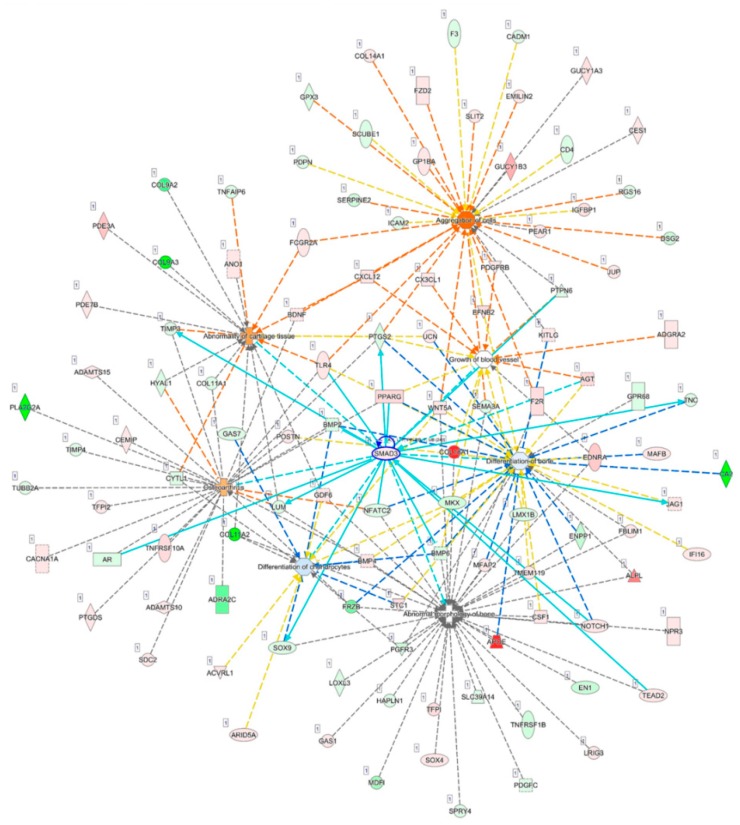
Analysis of interconnections and the merged network of OA-related diseases and functions. The 495 differentially expressed genes identified in OA knee chondrocytes were analyzed in the Ingenuity Pathway Analysis (IPA) software, and diseases and functions related to cellular, tissue and structural changes of cartilage in OA, including osteoarthritis, abnormality of cartilage tissue, differentiation of chondrocytes, growth of blood vessel, and aggregation of cells, were selected to identify target genes involved. *SMAD3* was centered to the merged network and interconnected with the molecules of other networks, as indicated in light blue lines, including those in the growth of the blood vessel. *WNT5A* was involved in aggregation of cells, which was possibly associated with the formation of chondrocyte clusters in OA.

Diseases and functions related to cellular, tissue and structural changes of cartilage in OA analyzed in the IPA software were selected, including osteoarthritis (*p* = 5.53 × 10^−11^, Z score = 0.762), abnormality of cartilage tissue (*p* = 5.06 × 10^−7^, Z score = 1.000), differentiation of chondrocytes (*p* = 6.60 × 10^−8^, Z score = −0.265), growth of blood vessel (*p* = 4.29 × 10^−7^, Z score = 0), and aggregation of cells (*p* = 1.15 × 10^−9^, Z score = 2.262); the merged network of these diseases and functions is shown in Figure 4. Among the seven significantly dysregulated genes identified in the previous section, we found that *SMAD3* was centered to the merged network, and interconnected with molecules of other networks, including those in the growth of blood vessel, a feature of cartilage damage in OA [9]. In addition, *WNT5A* was involved in the aggregation of cells related to the formation of chondrocyte clusters in OA [7,9,30].

### 3.5. Identification of Differentially Expressed miRNAs and Potential miRNA–mRNA Interactions between Normal and OA Knee Chondrocytes

**Figure 5:**
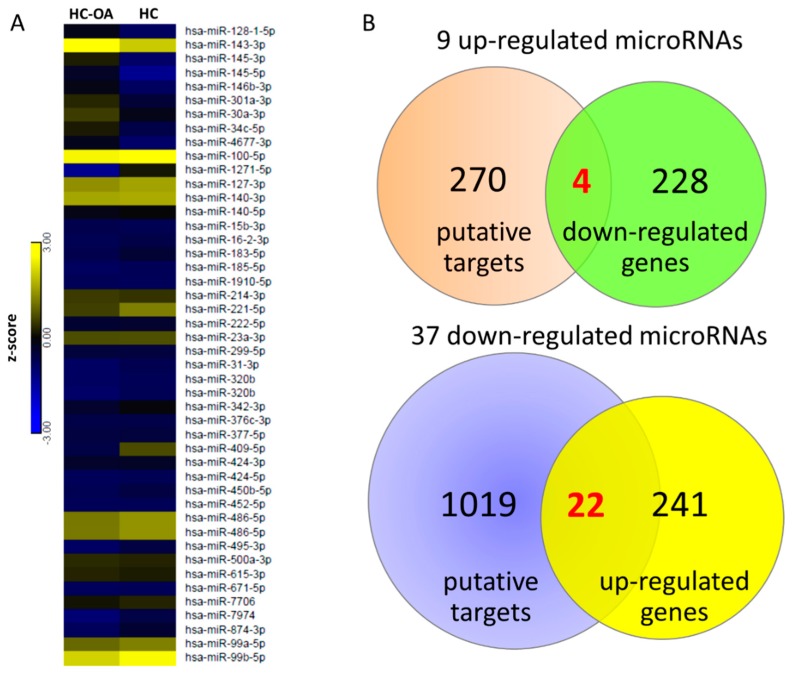
Identification of dysregulated miRNAs and potential miRNA–mRNA interactions in OA knee chondrocytes. (**A**) The 46 differentially expressed miRNAs of OA chondrocytes (>2.0-fold change and reads per million (RPM) > 10) were identified from the next-generation sequencing (NGS) result, and the heat map with z-score values is shown. (**B**) The miRmap database analysis identified 274 putative targets of nine up-regulated miRNAs and 1041 putative targets of 37 down-regulated miRNAs (selection threshold set at miRmap score ≥ 99.0). In addition, 232 down-regulated genes and 263 up-regulated genes (>2.0-fold change and fragments per kilobase of transcript per million (FPKM) > 0.3) in OA chondrocytes were identified from the NGS results. The putative targets of up-regulated (down-regulated) miRNAs were matched to our down-regulated (up-regulated) protein-coding genes using the Venn diagram analysis, which yielded four down-regulated genes and 22 up-regulated genes with potential miRNA–mRNA interactions in OA knee chondrocytes.

To determine the potential miRNA–mRNA interactions between normal and OA knee chondrocytes, we also performed small RNA sequencing by NGS, and identified 46 differentially expressed miRNAs (>2.0-fold change and >10 RPM of either origin of chondrocytes), including nine up-regulated and 37 down-regulated miRNAs in OA knee chondrocytes compared to normal chondrocytes. The detailed information of the 46 differentially expressed miRNAs is provided in Table S4. The heat map of the 46 differentially expressed miRNAs with z-score values is shown in Figure 5A. The putative targets of the 46 miRNAs were predicted using miRmap database and selected targets with miRmap score >99.0. The analytical results yielded 274 putative targets of nine up-regulated miRNAs and 1041 putative targets of 37 down-regulated miRNAs. The 274 targets of up-regulated miRNAs and 1041 targets of down-regulated miRNAs were then matched to our 232 down-regulated genes and 263 up-regulated genes obtained from high-throughput RNA sequencing, respectively. Using the Venn diagram analysis available on the website [31], four down-regulated genes and 22 up-regulated genes with potential miRNA-mRNA interactions in OA chondrocytes were identified, as depicted in Figure 5B.

### 3.6. Analysis of Candidate Genes with Potential miRNA-mRNA Interactions in Gene Expression Omnibus (GEO) Database and Identification of Potential Molecular Signatures in OA Knee Joint Microenvironment

**Figure 6:**
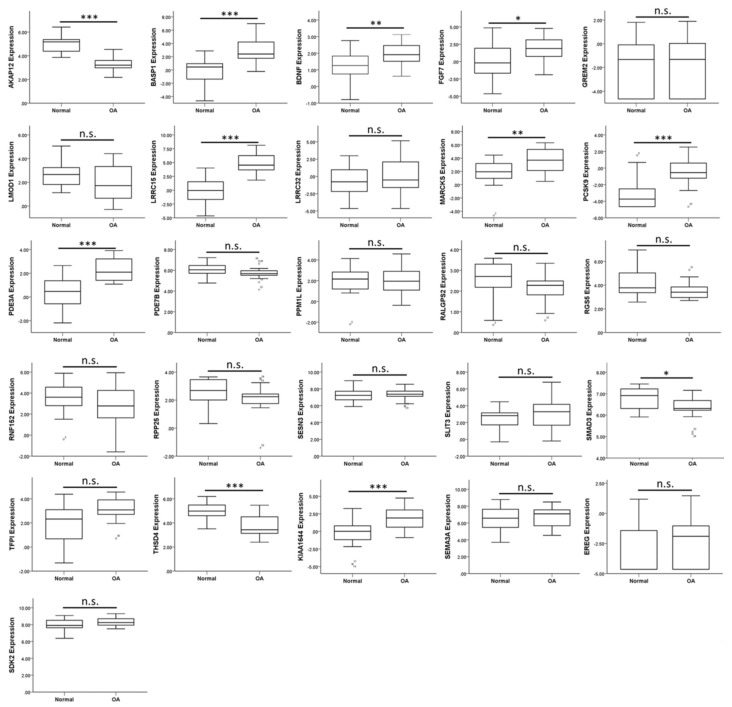
Analysis of 26 candidate genes with potential miRNA–mRNA interactions in the OA-related dataset. The expression values of 22 up-regulated and four down-regulated genes were analyzed in the knee cartilage dataset from GEO database (GSE114007). The significantly up-regulated expression patterns of *BASP1*, *BDNF*, *FGF7*, *LRRC15*, *MARCKS*, *PCSK9*, and *PDE3A* in OA knee cartilage were consistent with our OA chondrocytes NGS result. * indicated *p* < 0.05, ** indicated *p* < 0.01, *** indicated *p* < 0.001, and n.s. indicated no statistical significance between normal and OA groups.

The 26 candidate genes with potential miRNA–mRNA interactions are listed in Table 3. We searched in the GEO database for OA-related datasets to further determine the expression patterns of these candidate genes in clinical specimen. Since OA is considered a disease involving all joint tissues, we took datasets of cartilage, subchondral bone and synovium into consideration while searching for related datasets. There were two representative arrays analyzing synovial tissues of non-arthritis and OA patients (GSE55235 and GSE55457). Along with the datasets of cartilages and subchondral bones from knee OA patients used in the previous section (GSE114007 and GSE51588), the expression patterns of the 26 candidate genes were sequentially analyzed in these four representative datasets to determine genes potentially dysregulated in the OA microenvironment, and are summarized in Table 4. The up-regulated *MARCKS* was found up-regulated in OA knee cartilages, subchondral bones of OA tibial plateaus and one of the OA synovial tissue arrays, while the down-regulated *EREG* was found down-regulated in subchondral bones of OA tibial plateaus and one of the OA synovial arrays. Additionally, *LRRC15* was up-regulated in the OA knee cartilages, synovial tissues and the subchondral bones of medial, but not lateral tibial plateau. The expression patterns of the 26 candidate genes in the OA knee cartilage dataset (GSE114007) is shown in Figure 6.

### 3.7. Identification of Potential miRNA-mRNA Interactions of LRRC15, MARCKS, and EREG in OA Knee Chondrocytes

The consistent expression patterns of *LRRC15*, *MARCKS*, *PDE3A*, and *EREG* found in our NGS data and datasets of OA knee cartilages, subchondral bones and synovial tissues were analyzed in the miRmap database for potential miRNA regulations of these genes. Those potential miRNA regulations of *LRRC15*, *MARCKS*, *PDE3A*, and *EREG* with miRmap score >99.0 were selected, and matched to the 46 differentially expressed miRNAs identified from our miRNA data. The putative 3’UTR binding sites and sequences of the potential miRNA regulations identified from miRmap database were further validated in two other miRNA prediction databases, TargetScan and miRDB, and the results are listed in Table 5. There was one target binding site for miR-140-3p in the 3’UTR of *MARCKS* (Figure S3) and two target binding sites for miR-301a-3p in the 3’UTR of *EREG* (Figure S4) validated in all three miRNA prediction databases. However, no validated target binding site for miR-140-5p in the 3’UTR of *LRRC15* was found. In addition, the target binding site for miR-495-3p in the 3’UTR of *PDE3A* was validated in miRDB, but not in the TargetScan database.

### 3.8. MARCKS and EREG were Potentially Involved in the Pathogenesis of Arthritic Knee Joint Pain

**Figure 7:**
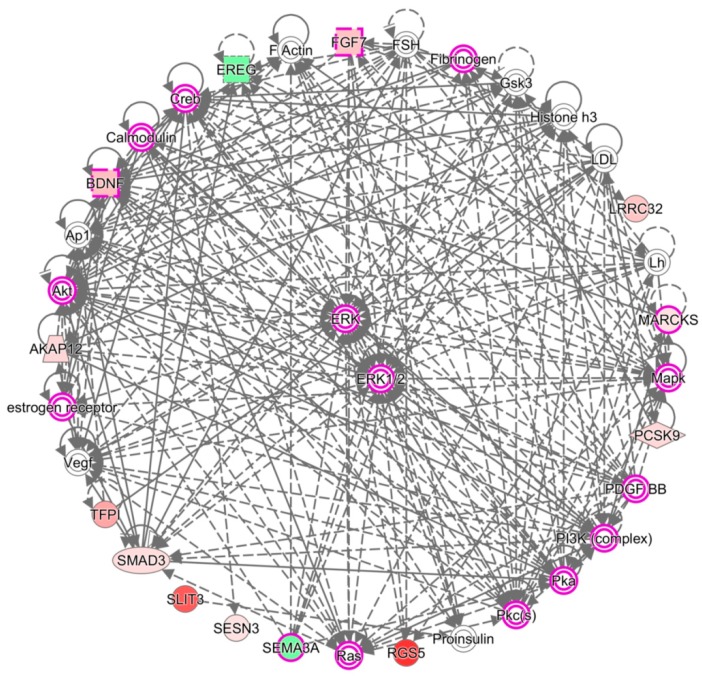
Network analysis by IPA identified molecules associated with the outgrowth of neurite. The network analysis of the 26 candidate genes by IPA software yielded three networks, where 13 genes were grouped into network 1, and are related to cellular movement, cardiovascular system development and function, and organismal development. The purple frames indicate molecules associated with the outgrowth of neurite, with four of our candidate genes, *BDNF*, *FGF7*, *MARCKS*, and *SEMA3A* potentially involved. Molecules with a green background indicate down-regulated expressions, and molecules with a red background indicate up-regulated expressions in OA knee chondrocytes compared to normal chondrocytes. The color scales indicate the relative expression values of OA to normal chondrocytes.

The 26 candidate genes were uploaded to the DAVID database and IPA software to disclose biological functions related to these differentially expressed genes of OA knee chondrocytes. The functional annotation analysis performed in the DAVID database identified 11 biological functions and their related genes; among them, three were directly related to nerve function, including axon extension, axon guidance, and synapse assembly (Table 6). The IPA network analysis of these genes yielded three categorized networks, as listed in Table 7. Network 1 related to cellular movement, cardiovascular system development and function, and organismal development possessed the highest score among the three networks, with 13 of the 26 genes involved in this network. The interconnection of molecules in network 1 is shown in Figure 7, with *MARCKS* and *EREG* in the same network. The overlay diseases and functions analysis identified *BDNF*, *FGF7*, *MARCKS*, and *SEMA3A* to be associated with “outgrowth of neurite”, as indicated by the purple frame in Figure 7.

**Figure 8:**
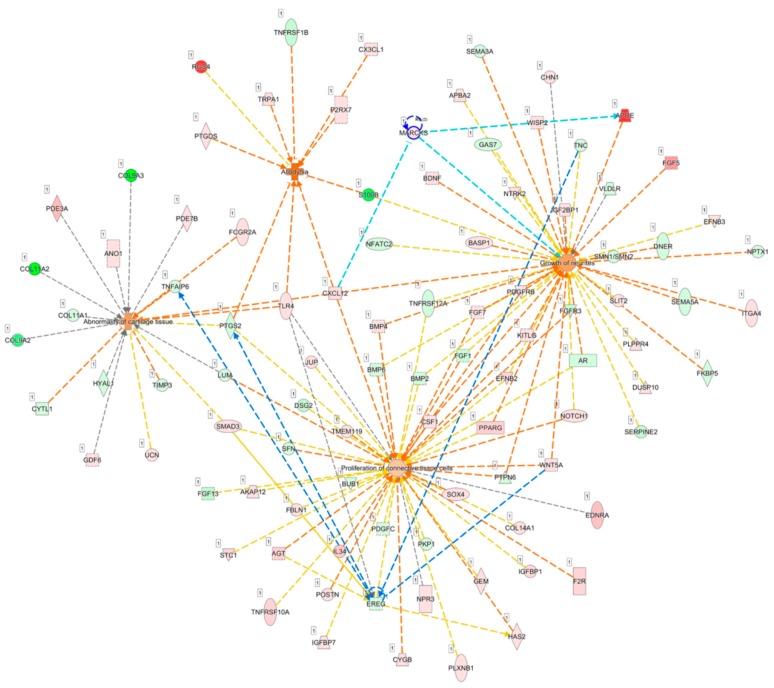
Analysis of the potential connections between *MARCKS*, *EREG* and OA joint pain condition. The merged network of genes related to the abnormality of cartilage tissue, proliferation of connective tissue cells, growth of neurites, and allodynia categorized from differentially expressed genes of OA knee chondrocytes by IPA software is shown. *MARCKS* was connected to *APOE* and *CXCL12*, as indicated in light blue lines, where *CXCL12* was demonstrated to be simultaneously involved in all four networks. *EREG* was mainly linked to *WNT5A*, *TNC*, *PTGS2*, and *TNFAIP6*, as indicated with blue lines.

Since *MARCKS* was associated with the outgrowth of neurite, we further explored the potential roles of *MARCKS* and *EREG* on the pain condition of OA. Diseases and functions involved in the differentially expressed genes of OA knee chondrocytes were categorized using the IPA software, and networks of genes related to abnormality of cartilage tissue (*p* = 5.06 × 10^−7^, Z score = 1.000), proliferation of connective tissue cells (*p* = 1.96 × 10^−9^, Z score = 0.512), growth of neurites (*p* = 6.40 × 10^−11^, Z score = 0.918), and allodynia (*p* = 1.68 × 10^−7^, Z score = 2.547) were merged to identify the connections between *MARCKS*, *EREG* and other differentially expressed genes (Figure 8). Using the “Connect” tool in the IPA, *MARCKS* was connected to *APOE* and *CXCL12* in this merged network, while *CXCL12* was one of the molecules involved in allodynia. In addition, *EREG* was linked to *WNT5A*, *TNC*, *PTGS2*, and *TNFAIP6*.

## 4. Discussion

In the current study, we identified dysregulated genes related to OA joint damage from primary OA knee chondrocytes to have similar expression patterns observed in the datasets of cartilages and subchondral bones of OA knees (GSE114007 and GSE51588). Through NGS and bioinformatics analysis, we also identified 46 differentially expressed miRNAs and 26 candidate genes potentially involved in OA knee chondrocytes. Of the 26 candidate genes, *MARCKS*, *EREG*, *LRRC15*, and *PDE3A* exerted similar expression patterns as observed in the datasets of cartilages, subchondral bones and synovial tissues of OA patients. Two potential miRNA–mRNA interactions were identified, including miR-140-3p-*MARCKS* and miR-301a-3p-*EREG*, and were validated systematically. These novel miRNA regulations are proposed to play critical roles in the pathogenesis of the altered OA knee joint microenvironment and to contribute partly to arthritic joint pain.

The close anatomical contact of articular cartilage to subchondral bone, altered bone structure correlating to aggravated cartilage degeneration, increased vascularization of the bone-cartilage interface observed during OA progression, and similar presentation of epigenetic phenotypes of eroded subchondral bone and cartilage in OA suggest potential crosstalk and molecular transport between cartilage, subchondral bone and the joint microenvironment [32,33,34]. The emerging role of extracellular vesicles in cartilage homeostasis and arthritis potentiated the possible mechanism of cell–cell communication between joint tissues through carriage of bioactive signaling molecules, such as miRNAs by extracellular vesicles [35,36,37]. Increasing evidence suggested extracellular vesicles released from chondrocytes may participate in dysregulated cartilage mineralization and contribute to OA [35,38]. Our study results identified *SMAD3* to potentially play a central role in the merged network of diseases and functions related to cellular, tissue and structural changes in OA, and serves as one of the molecules in the network of “growth of vessel” (Figure 4). In the OA joint, articular cartilage loses resistance to vascularization, and the growth of blood vessels increased particularly at the bone–cartilage interface [39]. TGF-β/SMAD2/3 signaling was shown to exert a chondroprotective effect in excessive mechanical compression by blocking chondrocyte differentiation [40]. Conversely, other studies indicated TGF-β/SMAD3 negatively regulated miR-140 in OA chondrocytes [41], and SMAD3 was significantly up-regulated in human OA cartilage tissue [42]. Additionally, TGF-β/Smad3 treatment enhanced vascular endothelial growth factor expression and inhibited vascular smooth muscle apoptosis through autocrine signaling [43]. These results suggested that TGF-β/Smad3 may have dual roles in the vascularization of OA cartilage and the development of OA.

In a review article of WNT5A signaling, the induced WNT5A activates non-canonical Wnt signaling to regulate the control of tissue polarity and cell aggregation and canonical WNT signaling for the proliferation of human synovial fibroblasts in rheumatoid arthritis (RA) [44]. In addition, the Wnt/β-catenin signaling is suggested to participate in the degradation of ECM in the degenerative joint of animal models by stimulating matrix catabolic genes and activity in the articular chondrocytes [45]. Recent studies also presented the positive correlation of the expression of Wnt5a in human articular cartilage to the progression of knee OA [46], and showed that Wnt5a promotes MMP production in human chondrocyte through non-canonical Wnt signaling [47]. The up-regulated WNT signaling in the inflammatory response of human chondrocytes was also proposed [48]. In the OA joint, chondrocytes aggregate to form clusters around the bone–cartilage interface [30]. Our current study identified the up-regulated *WNT5A* in OA knee chondrocytes, and bioinformatics analysis suggested the involvement of *WNT5A* in the positive regulation of cartilage development and the aggregation of cells, suggesting the important role of *WNT5A* in cartilage degradation in knee OA.

The differentially expressed miRNAs in the cartilage and bone of human knee OA with potential target genes related to inflammatory processes were identified [49]. Using the NGS and bioinformatics analysis, we identified the potential interaction of miR-140-3p-*MARCKS* in OA knee chondrocytes. Literature has identified miR-140 as a major contributor to cartilage homeostasis, targeting genes involved in the differentiation of chondrocytes [10,50]. Miyaki et al. reported the increased expression of miR-140 during chondrogenesis in human mesenchymal stem cells, the reduced expression of miR-140 in OA cartilage and in chondrocytes in response to IL-1β, and confirmed the dual roles of miR-140 in cartilage development and homeostasis in transgenic mouse model [51,52]. In a recent study analyzing the expressions of miR-140-3p and miR-140-5p in synovial fluid of OA and non-OA patients, both miRNAs were down-regulated in OA synovial fluid and were associated with the severity of OA [53]. The down-regulated miR-140-3p and miR-140-5p were also observed in our small RNA sequencing result of OA knee chondrocytes, indicating the potential target of miR-140 as biomarkers in knee OA.

Pain is a major symptom in patients with OA. The mechanisms involved in arthritic joint pain are complicated, while structural pathologies, neuronal mechanisms of pain, and general factors such as obesity and genetic factors shall all take part in the consequence of joint pain [54]. Central and peripheral sensitizations of the nociceptive system are extensively proposed mechanisms of neuronal causes of OA joint pain [55]. Normal articular cartilage lacks vascular supply and sensory innervation [56]; structural changes of innervation in OA may be potential contributors to joint pain, with an evident increase in nerve growth and vascular penetration to the meniscus [57,58]. Suri et al. also reported the presence of nerve fibers within vascular channels of OA cartilage and subchondral bone marrow, which may contribute to pain in knee OA [33]. In our current study, we identified the increased expression of *MARCKS* in OA knee chondrocytes, and observed the similar expression patterns of *MARCKS* in OA subchondral bones and synovial tissues. Myristoylated alanine-rich protein kinase C substrate (MARCKS) is an actin-binding protein distributed in the nervous system and is localized in axons and dendrites; its phosphorylation is involved in the release of neurotransmitters and synaptic trafficking, mechanisms related to inflammatory pain [59]. Bradykinin is a peptide involved in inflammation, with its B2 receptors expressed on various OA-related cell types, implicating its involvement in OA pathogenesis [60]. Tanabe et al. also reported a novel bradykinin signaling cascade of neurite outgrowth through MARCKS phosphorylation in a neuroblastoma cell line [61]. Studies also suggested local release of neurotrophins by non-neuronal tissues in inflammatory joint lining that facilitates nerve ingrowth into tissues [62], and released neurotrophins may exert autocrine/paracrine effects that regulate articular chondrocyte metabolism [63]. This provides clues to the connection between neurite outgrowth and the pathogenesis of arthritic joint pain. There is emerging evidence regarding the possible regulatory function of miRNAs in nociceptive pathways and it is a potential therapeutic target in OA management [12]. Our current results identified the potential role of miR-140-3p-*MARCKS* regulation in the OA knee joint microenvironment. Whether targeting this novel miRNA regulation assists in the management of joint pain or not, and whether it takes part in the altered structural innervation or peripheral sensitization in knee OA merits further investigation.

Another novel miRNA regulation of miR-301a-3p-*EREG* was identified in our current study. MiR-301a-3p is a pro-inflammatory miRNA that promotes inflammation during tumorigenesis [64]. There is not much literature reporting the role of miR-301a-3p in the regulation of arthritis. Two recent studies reported the regulation of miR-301a-3p in inflammatory response of RA and chondrocytes. In patients with RA, over-expressed miR-301a-3p was found in the peripheral blood mononuclear cells and it was positively correlated to an increased proportion of a subset of helper T cell [65]. Chen et al. studied the role of miR-301a in a mouse chondrogenic cell line in response to inflammatory injury using lipopolysaccharide (LPS), and reported the increased expression of miR-301a with LPS induction and the suppression of miR-301a alleviated LPS-induced chondrogenic cell injury by targeting Sirt1, implicating the potential therapeutic target of miR-301a for OA [66]. The down-regulated *EREG* in our NGS result was the putative target of miR-301a-3p. Epiregulin (EREG), an EGFR ligand, is expressed in various tissues; it plays important roles in wound healing and vascular remodeling during inflammation and regulates cell proliferation and differentiation [67]. A recent study by Martin et al. demonstrated the genetic association of *EREG* and *EGFR* with chronic pain in patients with temporo-mandibular joint disorder, indicating the role of EREG in pain processing [68]. However, the association of *EREG* expression in the joint microenvironment remains unclear. Only one report demonstrated the increased expression of *EREG* and pro-inflammatory cytokines in fibroblast-like synoviocytes, suggesting the role of *EREG*-mediated EGFR signaling to the pathogenesis of RA [69]. In our current study, we observed the similarly down-regulated *EREG* in OA knee chondrocytes, subchondral bone and synovial tissue of OA patients. Further investigation into the role of miR-301a-3p-*EREG* regulation in OA may provide further insight into better understanding novel miRNA regulation in the pathogenesis of knee OA.

**Figure 9:**
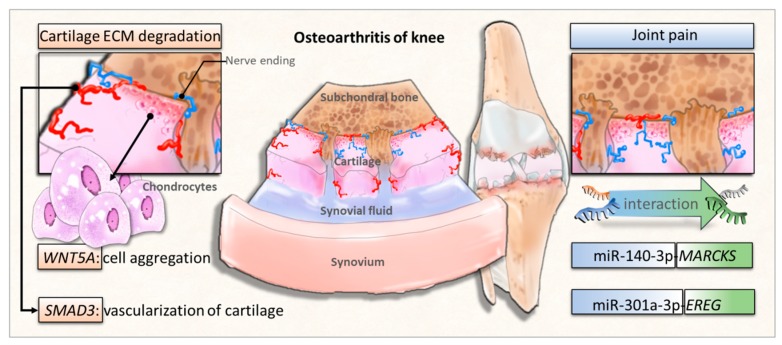
The proposed novel molecular signatures and miRNA regulations in osteoarthritic knee chondrocytes.

The characteristic change of OA is cartilage breakdown, but a growing consensus has proposed OA as a disease of the whole joint, involving all joint tissues [9]. In the current study, we identified several important molecules involved in the structural damage of OA knee joint and explored novel miRNA–mRNA regulations potentially involved in the pathogenesis of OA joint pain. The schematic potential molecular signatures and miRNA regulations related to OA structural damage and joint pain are summarized in Figure 9. These identified novel miRNA–mRNA regulations may provide new insights into the potential targets for arthritic joint pain. Further validation with experimental studies on clinical OA knee cartilage specimens are necessary to consolidate the clinical significance of the current *in silico* results.

## 5. Conclusions

Our current study identified the critical roles of *SMAD3* and *WNT5A* in the OA knee joint microenvironment, which is potentially linked to features of ECM degradation in knee OA. Additionally, miR-140-3p-*MARCKS* and miR-301a-3p-*EREG* regulations were potentially involved in the pathogenesis of the altered OA knee joint microenvironment. The current findings suggest new perspectives in studying novel genes potentially contributing to arthritic joint pain in knee OA, which may assist in finding new targets for OA treatment.

## Figures and Tables

**Table 1:** Top canonical pathways and upstream regulators involved in differentially expressed genes of osteoarthritic (OA) knee chondrocytes.

Category	No. of Genes	*p*-Value
Top canonical pathways		
Osteoarthritis Pathway	23	4.72 × 10^−10^
Hepatic Fibrosis/Hepatic Stellate Cell Activation	20	8.99 × 10^−9^
Axonal Guidance Signaling	31	4.32 × 10^−8^
Atherosclerosis Signaling	15	1.77 × 10^−7^
GP6 Signaling Pathway	13	1.06 × 10^−5^
Top predicted upstream regulators		
*TNF*		2.20 × 10^−22^
*TGFB1*		1.01 × 10^−21^
Dexamethasone		1.57 × 10^−16^
*IFNG*		6.75 × 10^−16^
*CTNNB1*		1.50 × 10^−15^

*CTNNB1*, catenin beta 1; *IFNG*, interferon gamma; *TGFB1*, transforming growth factor beta 1; *TNF*, tumor necrosis factor.

**Table 2:** Selected biological functions related to cellular, tissue and structural changes in osteoarthritis.

Biological Process	Count	*p*-Value	Up-Regulated Genes	Down-Regulated Genes	Fold Enrichment
Positive regulation of bone mineralization	7	2.66 × 10^−4^	*BMP4, GPM6B, P2RX7, SMAD3, TMEM119*	*BMP2, BMP6*	7.62
Positive regulation of chondrocyte differentiation	5	1.32 × 10^−3^	*ACVRL1, GDF6,SMAD3*	*BMP6, SOX9*	10.02
Positive regulation of cartilage development	4	7.76 × 10^−3^	*BMP4, WNT5A*	*BMP2, SOX9*	9.52
Chondrocyte differentiation	5	1.86 × 10^−2^	*BMP4*	*BMP2, COL11A2, CYTL1, FGFR3*	4.88
Endochondral ossification	4	2.97 × 10^−2^	*ALPL, BMP4*	*BMP6, FGFR3*	5.86

**Table 3:** Candidate genes of OA knee chondrocytes identified from putative targets of miRNAs.

Gene Symbol	Gene Name	HC-OA FPKM	HC FPKM	Fold-Change (HC-OA/HC)
*AKAP12*	A-kinase anchoring protein 12	94.69	32.02	2.96
*BASP1*	brain abundant, membrane attached signal protein 1	80.88	38.09	2.12
*BDNF*	brain-derived neurotrophic factor	8.28	1.76	4.70
*FGF7*	fibroblast growth factor 7	37.21	8.52	4.37
*GREM2*	gremlin 2, DAN family BMP antagonist	9.66	3.54	2.73
*LMOD1*	leiomodin 1	26.03	1.78	14.65
*LRRC15*	leucine rich repeat containing 15	6.47	2.56	2.53
*LRRC32*	leucine rich repeat containing 32	9.64	2.30	4.19
*MARCKS*	myristoylated alanine-rich protein kinase C substrate	278.54	133.99	2.08
*PCSK9*	proprotein convertase subtilisin/kexin type 9	1.51	0.53	2.86
*PDE3A*	phosphodiesterase 3A	19.30	1.88	10.25
*PDE7B*	phosphodiesterase 7B	4.98	1.53	3.27
*PPM1L*	protein phosphatase, Mg2+/Mn2+ dependent 1L	4.44	0.97	4.59
*RALGPS2*	Ral GEF with PH domain and SH3 binding motif 2	48.94	18.20	2.69
*RGS5*	regulator of G-protein signaling 5	90.50	6.24	14.50
*RNF152*	ring finger protein 152	15.71	3.83	4.10
*RPP25*	ribonuclease P/MRP 25kDa subunit	4.69	2.02	2.32
*SESN3*	sestrin 3	14.57	6.95	2.10
*SLIT3*	slit guidance ligand 3	29.08	2.46	11.80
*SMAD3*	SMAD family member 3	408.29	166.01	2.46
*TFPI*	tissue factor pathway inhibitor	56.10	8.72	6.43
*THSD4*	thrombospondin type 1 domain containing 4	3.44	0.65	5.31
*KIAA1644*	KIAA1644	22.55	61.04	0.37
*SEMA3A*	semaphorin 3A	12.52	39.96	0.31
*EREG*	epiregulin	1.50	4.65	0.32
*SDK2*	sidekick cell adhesion molecule 2	2.04	15.99	0.13

HC-OA, OA knee chondrocytes; HC, normal knee chondrocytes; FPKM, fragments per kilobase of transcript per million mapped reads.

**Table 4:** Analysis of 26 candidate genes in osteoarthritis-related datasets in Gene Expression Omnibus database.

GEO Accession Number	GSE114007	GSE51588		GSE55235	GSE55457
Specimen	Cartilage	Subchondral bone		Synovial tissue	
	Normal/OA	Normal/OA		Normal/OA	Normal/OA
		Medial	Lateral		
Numbers	18/20	5/20	5/20	10/10	10/10
Up-Regulated mRNA *					
*AKAP12*	DOWN	n.s. ^†^	n.s.	n.s.	n.s.
*BASP1*	UP	n.s.	DOWN	n.s.	n.s.
*BDNF*	UP	n.s.	n.s.	n.s.	n.s.
*FGF7*	UP	n.s.	n.s.^†^	n.s.	n.s.
*GREM2*	n.s.	n.s.	n.s.	n.s.	n.s.
*LMOD1*	n.s.	n.s.	n.s.	DOWN	n.s.
***LRRC15***	**UP**	**UP**	n.s.	**UP**	**UP**
*LRRC32*	n.s.	n.s.	n.s.	n.s.	n.s.
***MARCKS***	**UP**	**UP**	**UP**	**UP**	n.s. ^†^
*PCSK9*	UP	UP	n.s.	--	--
***PDE3A***	**UP**	**UP**	**UP**	n.s.	n.s.
*PDE7B*	n.s.	n.s.	n.s.	DOWN	n.s.
*PPM1L*	n.s.	n.s.	n.s.	--	--
*RALGPS2*	n.s.	n.s.	n.s.	n.s.	n.s.
*RGS5*	n.s.	n.s.	n.s.	n.s.	n.s.
*RNF152*	n.s.	n.s.	n.s.	--	--
*RPP25*	n.s.	UP	n.s.	n.s.	n.s.
*SESN3*	n.s.	n.s.	n.s.	--	--
*SLIT3*	n.s.	UP	n.s. ^†^	n.s. ^†^	n.s. ^†^
*SMAD3*	DOWN	UP	UP	n.s. ^†^	n.s. ^†^
*TFPI*	n.s.	n.s. ^†^	n.s.	DOWN	n.s.
*THSD4*	DOWN	UP	UP	n.s.	n.s.
Down-Regulated mRNA *					
*KIAA1644*	UP	n.s. ^†^	n.s.	UP	n.s.
*SEMA3A*	n.s.	n.s.	n.s.	UP	n.s.
***EREG***	n.s.	**DOWN**	**DOWN**	**DOWN**	n.s.
*SDK2*	n.s.	UP	UP	n.s.	n.s.

The genes and their expression patterns marked in **bold** indicated those with more consistent expression patterns in OA related datasets. * For those genes with more than one probe within the array, the expression patterns were considered consistent only if there were at least two probes with significant dysregulation in the same direction. UP, significantly up-regulated in OA (*p* < 0.05); DOWN, significantly down-regulated in OA (*p* < 0.05); n.s., non-significant between normal and OA. -- indicated no identical probes within the array. ^†^ indicated there were at least two probes for the gene and the expression of the gene was significant only in one of the probes.

**Table 5:** Potential miRNA regulations of four identified genes in OA knee chondrocytes.

**Down-Regulated miRNA**	**Fold-Change**	**Predicted Target** **Up-Regulated mRNA**	**miRmap Score**	**TargetScan**	**miRDB**
hsa-miR-140-5p	−3.11	*LRRC15*	99.03	−	−
hsa-miR-140-3p	−2.87	*MARCKS*	99.27	+	+
hsa-miR-495-3p	−4.80	*PDE3A*	99.93	−	+
**Up-Regulated miRNA**	**Fold-Change**	**Predicted Target** **Down-Regulated mRNA**	**miRmap Score**	**TargetScan**	**miRDB**
hsa-miR-301a-3p	2.45	*EREG*	99.06	+	+

**Table 6:** Biological functions of 26 candidate genes in OA knee chondrocytes.

Biological Process	*p*-Value	Related Genes	Fold Enrichment
Negative regulation of TORC1 signaling	0.013	*RNF152, SESN3*	149.26
Cytokine-mediated signaling pathway	0.015	*EREG, LRRC15, GREM2*	15.38
Axon extension involved in axon guidance	0.017	*SEMA3A, SLIT3*	111.95
cAMP catabolic process	0.021	*PDE7B, PDE3A*	89.56
Axon guidance	0.021	*BDNF, SEMA3A, SLIT3*	12.67
Oocyte maturation	0.025	*EREG, PDE3A*	74.63
Positive regulation of GTPase activity	0.045	*RALGPS2, FGF7, EREG, RGS5*	4.76
Negative chemotaxis	0.048	*SEMA3A, SLIT3*	39.51
MAPK cascade	0.053	*FGF7, EREG, PPM1L*	7.69
Positive regulation of cell division	0.065	*FGF7, EREG*	28.58
Synapse assembly	0.084	*BDNF, SDK2*	22.02

**Table 7:** Networks associated with 26 candidate genes differentially expressed in OA knee chondrocytes.

	Top Diseases and Functions	Score	Focus Molecules	Molecules in Network
1	Cellular Movement, Cardiovascular System Development and Function, Organismal Development	32	13	**↑AKAP12**, Akt, Ap1, **↑BDNF**, Calmodulin, Creb, **↓EREG**, ERK,ERK1/2, estrogen receptor, F Actin, **↑FGF7**, Fibrinogen, FSH, Gsk3, Histone h3, LDL, Lh, **↑LRRC32**, Mapk, **↑MARCKS**, **↑PCSK9**, PDGF BB, PI3K (complex), Pka, Pkc(s), Proinsulin, Ras, **↑RGS5**, **↓SEMA3A**, **↑SESN3**, **↑SLIT3**, **↑SMAD3**, **↑TFPI**, Vegf
2	Cardiovascular Disease, Organismal Injury and Abnormalities, Cardiovascular System Development and Function	15	7	26s Proteasome, BMP2, CLU, F10, G protein alphai, GAS6, **↑GREM2**, HNF4A, Jnk, LPA, LRPAP1, **↑LRRC15**, MAGI3, MAP2K5, MAP3K, MAPK9, mir-25, mir-181, MMP9, MMP12, MZB1, Neurotrophin, NFkB (complex), NRG (family), P38 MAPK, PP1/PP2A, Pp2c, **↑PPM1L**, PTPN13, **↑RALGPS2**, **↑RNF152**, SAA, **↓SDK2**, **↑THSD4**, tyrosine kinase
3	Gastrointestinal Disease, Hepatic System Disease, Organismal Injury and Abnormalities	12	6	ABCC4, AR, **↑BASP1**, C2CD5, CENPE, CENPH, CEPT1, EGR3, ELAVL1, GADD45GIP1, INPP5K, **↓KIAA1644**, KIF11, KIFC3, LMNB1, **↑LMOD1**, miR-149-3p, miR-185-5p, MRRF, NONO, Pde, **↑PDE3A**, PDE4A, PDE5A, **↑PDE7B**, PFKFB3, PITX3, PLBD2, PRMT1, PSMF1, Rb, RNF14, **↑RPP25**, TAF12, ZNF281

The genes marked in **bold** were the 26 candidate genes identified in osteoarthritic chondrocytes.

## Supplementary Materials

The following are available online at http://www.mdpi.com/2077-0383/7/12/535/s1, Table S1: Available information of selected osteoarthritis related datasets from GEO database, Table S2: RNA and small RNA sequencing summary, Table S3: The list of 495 differentially expressed genes in OA knee chondrocytes, Table S4: The list of 46 differentially expressed miRNAs in OA knee chondrocytes, Figure S1: RNA sequencing quality control report for normal adult chondrocytes (HC) and osteoarthritic knee chondrocytes (HC-OA), Figure S2: Small RNA sequencing quality control report for HC and HC-OA, Figure S3: The putative 3’UTR binding site of miR-140-3p on *MARCKS*, Figure S4: The putative 3’UTR binding site of miR-301a-3p on *EREG*.
