# Supplementary material for: Systematic Analysis of Transcriptomic Profile of Chondrocytes in Osteoarthritic Knee Using Next-Generation Sequencing and Bioinformatics
Source: J Clin Med. 2018 Dec 10;7(12):535. doi: 10.3390/jcm7120535 (PMC6306862; doi:10.3390/jcm7120535)
Supplement: Supplementary file 1 [file jcm-07-00535-s001.zip › jcm-379338-SI.pdf]

Table S1. Available information of selected osteoarthritis related datasets from GEO database.

**GSE114007**				
Knee cartilage	Normal		OA	
Number	18		20	
Age (years)	18–61 (mean age 38)		52–82 (mean age 66)	
Gender				
Male	13		8	
Female	5		12	
**GSE51588**				
Tibial plateau subchondral bone	Normal		OA	
	Lateral	Medial	Lateral	Medial
Number	5	5	20	20
Age (years)	38.40 ± 13.45	38.40 ± 13.45	70.15 ± 8.87	69.20 ± 9.03
Gender				
Male	2	2	12	10
Female	3	3	8	10
**GSE55457**				
Knee synovium	Normal		OA	
Number	10		10	
Age (years)	51.00 ± 19.71		72.40 ± 5.93	
Gender				
Male	8		2	
Female	2		8	
**GSE55235**				
Knee synovium	Normal		OA	
Number	10		10	

Table S2. RNA and small RNA sequencing summary.

<table>
<tr><th colspan="6">RNA Sequencing</th></tr>
<tr><td></td><td colspan="2">Total Reads after PF</td><td colspan="2">Total Reads after QT</td><td>Mappability</td></tr>
<tr><td>HC</td><td colspan="2">38445214</td><td colspan="2">36909105</td><td>88.11%</td></tr>
<tr><td>HC-OA</td><td colspan="2">38381030</td><td colspan="2">36908690</td><td>88.71%</td></tr>
<tr><th colspan="6">Small RNA Sequencing</th></tr>
<tr><td></td><td>Total Reads after PF</td><td>Total Reads after QT</td><td>Reads after processing</td><td>miRNA Reads</td><td>Non-miRNA Reads</td></tr>
<tr><td>HC</td><td>14493942</td><td>11997706</td><td>11671963</td><td>10005152</td><td>1666811</td></tr>
<tr><td>HC-OA</td><td>14474532</td><td>12546008</td><td>12346614</td><td>10945373</td><td>1401241</td></tr>
</table>

PF: Passing Filter; QT: Quality Trim; HC-OA, OA knee chondrocytes; HC, normal knee chondrocytes.

Table S3. The list of 495 differentially expressed genes in OA knee chondrocytes.

Table S4. The list of 46 differentially expressed miRNAs in OA knee chondrocytes.

* Table S3 and S4 are provided as separated supplementary file in excel format.

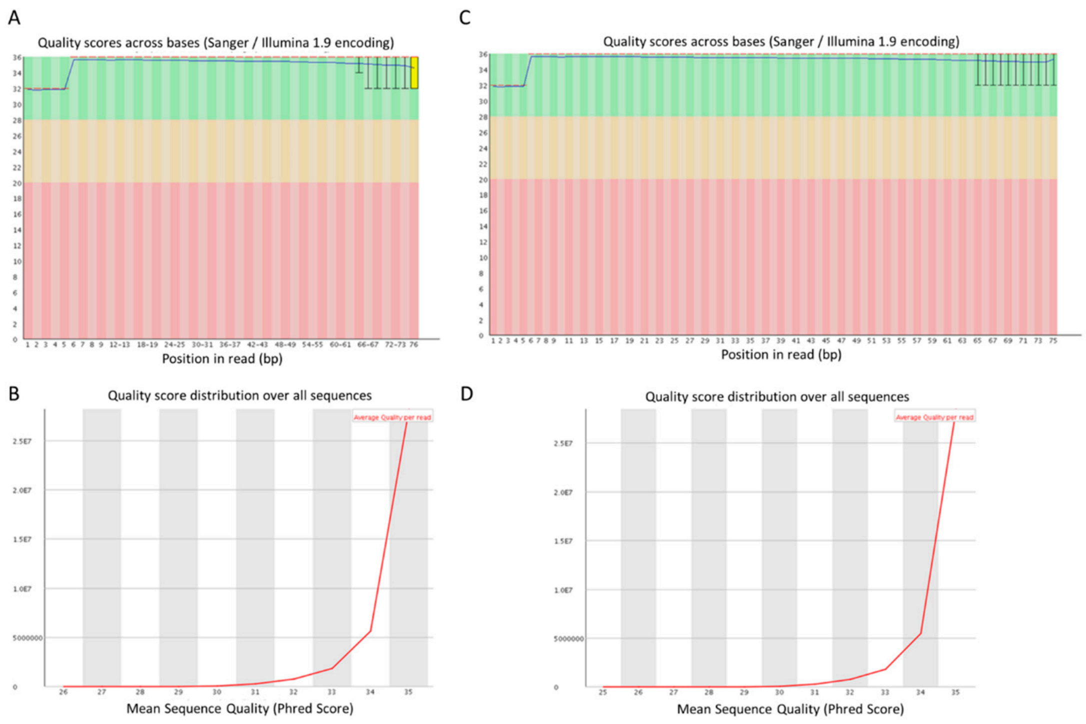

Figure S1. RNA sequencing quality control report for normal adult chondrocytes (HC) and osteoarthritic knee chondrocytes (HC-OA). The quality of sequencing result was determined by FastQC, reporting per base sequence quality and per sequence quality scores for HC (**A**, **B**) and HC-OA (**C**, **D**). The quality scores across all bases fell into the green background, indicating good quality calls. In addition, majority of reads were >35, indicating good sequencing quality.

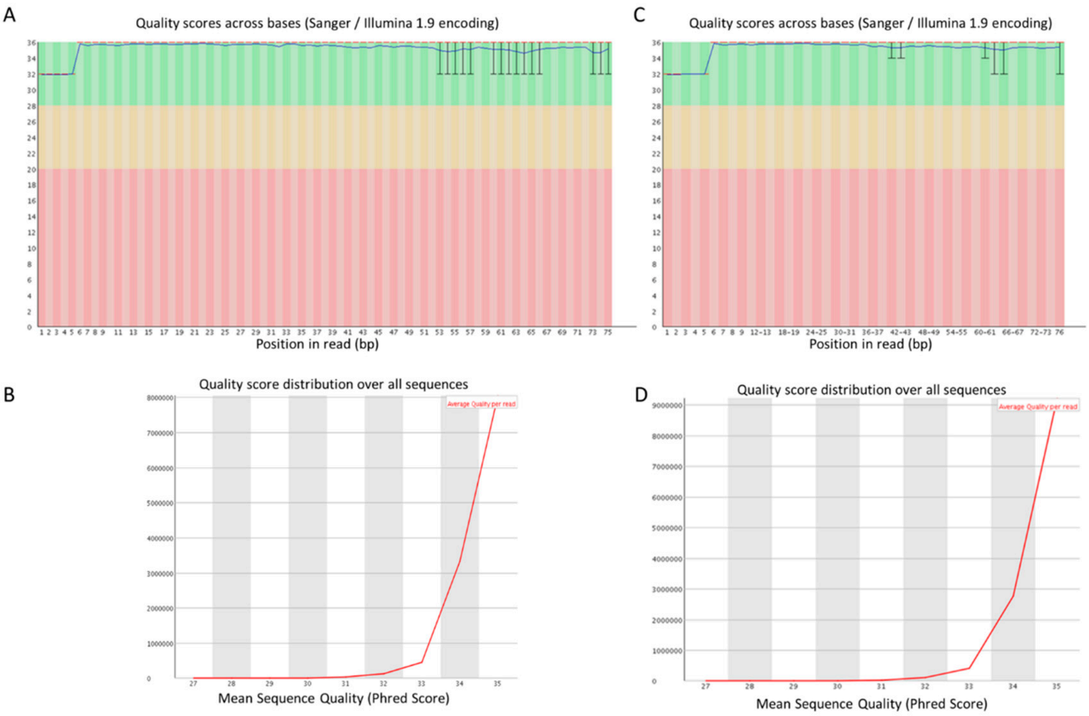

Figure S2. Small RNA sequencing quality control report for HC and HC-OA. The quality of sequencing result was determined by FastQC, a quality control tool for high-throughput sequence data, reporting per base sequence quality and per sequence quality scores for HC (**A**, **B**) and HC-OA (**C**, **D**). The quality scores across all bases fell into the green background, indicating good quality calls. In addition, majority of reads were >35, indicating good sequencing quality.

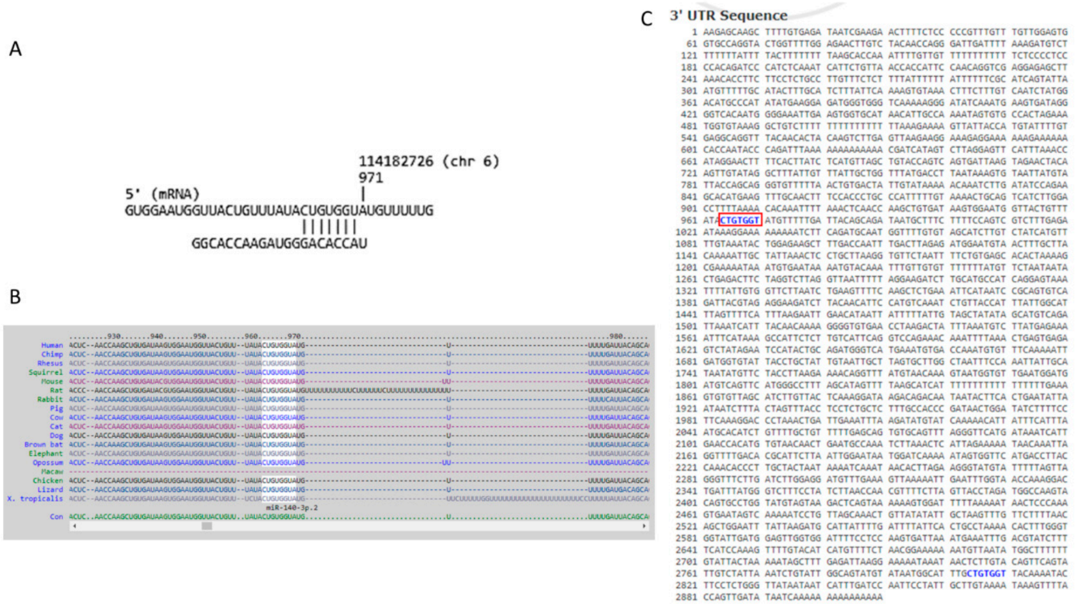

Figure S3. The putative 3′UTR binding site of miR-140-3p on *MARCKS*. The sequence and putative binding site of miR-140-3p on the 3′UTR of *MARCKS* at position of 964–970 was validated in miRmap (**A**); TargetScan (**B**); and miRDB (**C**).

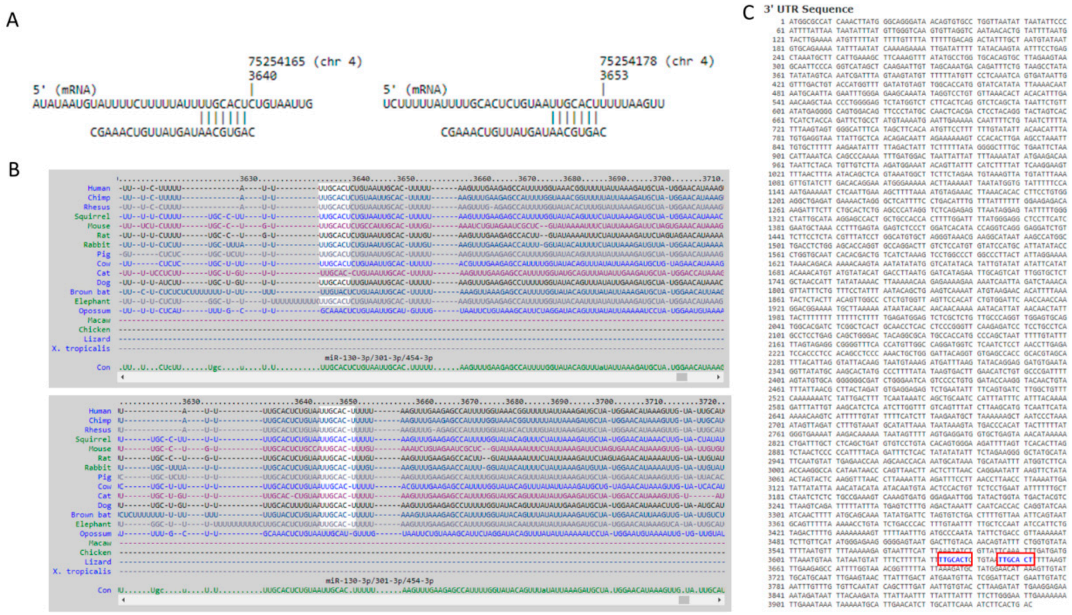

Figure S4. The putative 3′UTR binding site of miR-301a-3p on *EREG*. The sequences and putative binding sites of miR-301a-3p on the 3′UTR of *EREG* at positions of 3633–3639 and 3646-3652 were validated in miRmap (**A**); TargetScan (**B**); and miRDB (**C**).
